# Amide proton transfer magnetic resonance imaging in detecting intracranial hemorrhage at different stages: a comparative study with susceptibility weighted imaging

**DOI:** 10.1038/srep45696

**Published:** 2017-04-04

**Authors:** Xiaoyue Ma, Yan Bai, Yusong Lin, Xiaohua Hong, Taiyuan Liu, Lun Ma, E Mark Haacke, Jinyuan Zhou, Jian Wang, Meiyun Wang

**Affiliations:** 1 Department of Radiology, Zhengzhou University People’s Hospital & Henan Provincial People’s Hospital, Zhengzhou, Henan, China; 2 Cooperative Innovation Center of Internet Healthcare & School of Software and Applied Technology, Zhengzhou, Henan, China; 3 Department of Radiology, Johns Hopkins University School of Medicine, Baltimore, Maryland, USA; 4 Department of Radiology, Wayne State University, Detroit, Michigan, USA; 5 Department of Anesthesiology and Critical Care Medicine, Johns Hopkins University School of Medicine, Baltimore, Maryland, USA

## Abstract

Amide proton transfer (APT) imaging is a noninvasive molecular magnetic resonance imaging (MRI) technique based on the chemical exchange-dependent saturation transfer mechanism. The purpose of this study was to investigate the diagnostic performance of APT MRI in detecting intracranial hemorrhage (ICH) at hyperacute, acute and subacute stages by comparing with susceptibility weighted imaging (SWI). APT MRI and SWI were performed on 33 included patients with ICH by using a 3-T MRI unit. A two-sided Mann-Whitney *U* test was used to detect differences in APT-weighted (APTw) and SWI signal intensities of ICH at hyperacute, acute and subacute stages. Receiver operating characteristic analysis was used to assess the diagnostic utilities of APT MRI and SWI. Our results showed that APT MRI could detect ICH at hyperacute, acute and subacute stages. Therefore, APTw signal intensity may serve as a reliable, noninvasive imaging biomarker for detecting ICH at hyperacute, acute and subacute stages. Moreover, APT MRI could provide additional information for the ICH compared with SWI.

Intracranial hemorrhage (ICH) is a serious neurologic disease associated with high mortality and disability^1^,^2^. Accurate and early diagnosis is important for improving ICH patient outcome^3^. Computed tomography (CT) is widely used to detect ICH and is considered the gold standard for imaging ICH^4^. However, CT has limited capability for detecting the early cerebral ischemia^5^, which has symptoms that are similar to those of ICH^6^. Diffusion-weighted imaging (DWI) is the most sensitive magnetic resonance imaging (MRI) technique for the early detection of cerebral ischemia, whereas the appearances of ICH on DWI are complicated and easily disturbed by magnetic susceptibility effect^7^,^8^. Conventional MRI can display the hemorrhagic lesions, but the imaging manifestations are complicate because the components of hematoma vary with time.

Susceptibility weighted imaging (SWI) is an MRI technique that is sensitive to paramagnetic blood products such as deoxyhemoglobin, methemoglobin, hemosiderin and ferritin^9^,^10^. It is thought to be the most sensitive tool available for detecting cerebral microbleeds. However, SWI has limited value in the early detection of cerebral ischemia.

Amide proton transfer (APT) imaging is a noninvasive molecular MRI technique based on the chemical exchange-dependent saturation transfer mechanism^11^. The APT MRI contrast is generated by endogenous mobile protein and peptide concentrations or pH changes in biological tissues^12^,^13^. APT MRI has been promisingly applied to patients with hyperacute and acute ischemia in previous studies^14^,^15^. Our previous study using the rat models of strokes showed that APT MRI also has the capability for the early and simultaneous detection of ICH and cerebral ischemia^16^. However, to the best of our knowledge, no study of patients has yet confirmed the diagnostic capability of APT MRI in the early detection of ICH or compared APT MRI with SWI for the detection of ICH at different stages. The purpose of this study was to investigate the diagnostic performance of APT MRI in detecting ICH at hyperacute, acute and subacute stages by comparing with SWI.

## Results

### Patient groups

7 of 33 included patients (21%) had hyperacute ICH (time interval range, 1–8 hours; mean interval, 4 hours), 7 patients (21%) had acute ICH (time interval range, 16–70 hours; mean interval, 60 hours), and the remaining 19 patients (58%) had subacute ICH (time interval range, 96–715 hours; mean interval, 339 hours).

### A comparative analysis of APT MRI and SWI in identifying ICH

**Figure 1:**
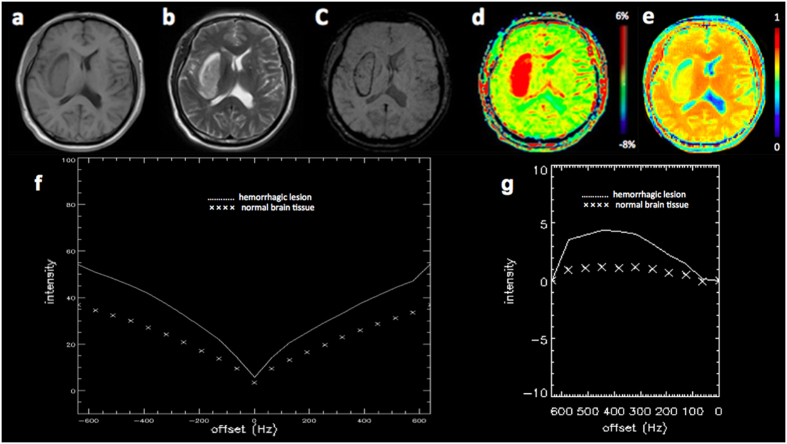
A 55-year-old male patient with hyperacute intracranial hemorrhage (ICH) (1 hours from stroke symptom onset) in the right basal ganglia. The hematoma shows mixed-signal on (**a**) T1w image and (**b**) T2w image. (**c**) Susceptibility weighted imaging (SWI) image shows an isointense center with a hypointense periphery. (**d**) Amide proton transfer (APT) image shows hyperintense signal. (**e**) MTR(3.5 ppm) image shows hypointense signal. (**f**) Z spectra map. (**g**) Z spectra asymmetry curve map.

**Figure 2:**
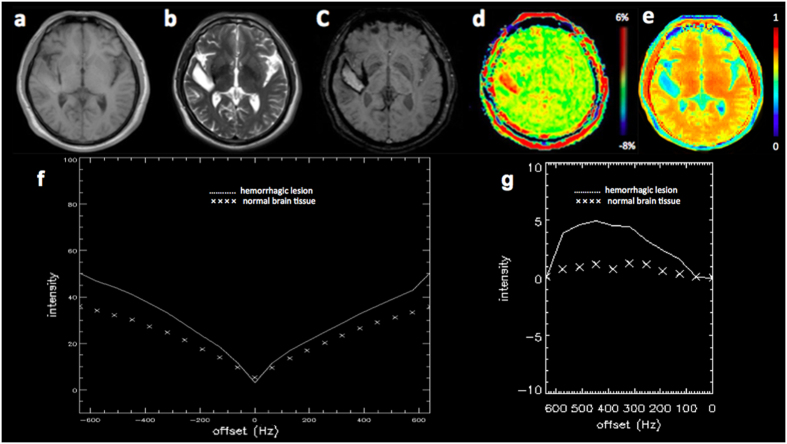
A 45-year-old male patient with acute ICH (39 hours from stroke symptom onset) in the right temporal lobe. The hematoma shows isointense signal on (**a**) T1w image and hyperintense signal on (**b**) T2w image. (**c**) SWI image shows a hyperintense center with a hypointense periphery. (**d**) APT image shows hyperintense signal. (**e**) MTR(3.5 ppm) image shows hypointense signal. (**f**) Z spectra map. (**g**) Z spectra asymmetry curve map.

**Figure 3:**
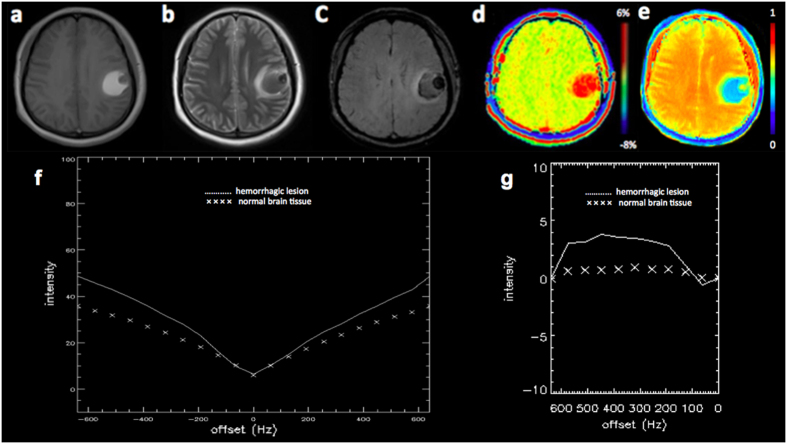
A 24-year-old female patient with subacute ICH (240 hours from stroke symptom onset) in the left parietal lobe. The hematoma shows mixed-signal on (**a**) T1w image, (**b**) T2w image and (**c**) SWI image. (**d**) APT image shows hyperintense signal. (**e**) MTR(3.5 ppm) image shows hypointense signal. (**f**) Z spectra map. (**g**) Z spectra asymmetry curve map.

Figures 1, 2 and 3 showed the imaging manifestations of ICH at hyperacute, acute and subacute stages, respectively. Table 1 quantitatively compared the APT-weighted (APTw), magnetization transfer ratio (MTR)(3.5 ppm) and SWI signal intensities between ICH at hyperacute, acute and subacute stages and the corresponding contralateral normal brain tissue. The APTw signal intensity was significantly higher in the ICH at hyperacute, acute and subacute stages than that in the contralateral normal brain tissue (all p < 0.001). The MTR(3.5 ppm) signal intensity was significantly lower in the ICH at hyperacute, acute and subacute stages than that in the contralateral normal brain tissue (p = 0.03, 0.01 and <0.001, respectively). The SWI signal intensity was significantly lower in the ICH at hyperacute and acute stages than that in the contralateral normal brain tissue (p = 0.03 and 0.02, respectively), but we observed no significant difference between the subacute ICH and the contralateral normal brain tissue (p = 0.11). After Bonferroni corrections for multiple comparisons, the APTw signal intensity was significantly higher in the hyperacute ICH than that in the subacute ICH (p = 0.014).

### Receiver operating characteristic (ROC) analysis for APT MRI and SWI in identifying ICH

**Figure 4:**
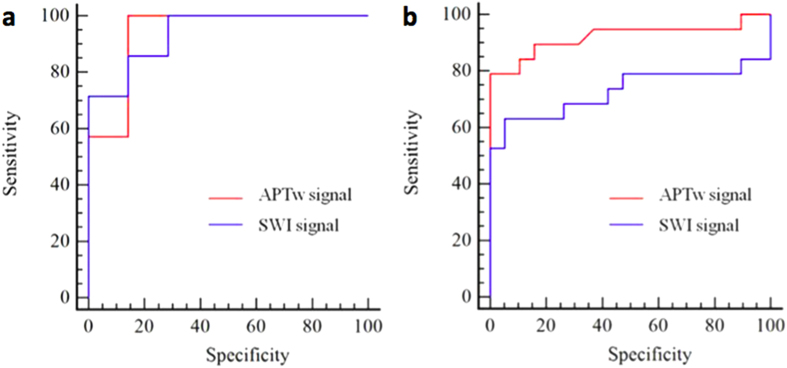
Receiver operating characteristic analysis. (**a**) The area under the receiver operating characteristic curves for the APTw signal intensity (0.94) and the SWI signal intensity (0.94) in differentiating the ICH at acute stage from the contralateral normal brain tissue. (**b**) The area under the receiver operating characteristic curve of the APTw signal intensity (0.92) is greater than that of the SWI signal intensity (0.73) in identifying the subacute ICH from the contralateral normal brain tissue (p = 0.02).

The areas under the ROC curves (AUCs) were analyzed as measures to differentiate the ICH at hyperacute, acute and subacute stages from the contralateral normal brain tissue. The AUC analysis yielded 1.00 for both APTw signal intensity and SWI signal intensity in identifying ICH at the hyperacute stage, and yielded 0.94 for both APTw signal intensity and SWI signal intensity in identifying ICH at the acute stage (Fig. 4a). The APTw and SWI signal intensities did not differ in their capability to differentiate the hyperacute and acute ICH from the contralateral normal brain tissue (p = 0.16). The AUC of the APTw signal intensity (0.92) was significantly greater than that of the SWI signal intensity (0.73) in identifying the subacute ICH from the contralateral normal brain tissue (p = 0.02) (Fig. 4b).

### Intraclass correlation coefficients

The intraclass correlation coefficients between two independent radiologists (** and **, with 9 and 18 years of experiences, respectively) for the calculations of APTw, MTR(3.5 ppm) and SWI signal intensities were 0.87, 0.82 and 0.84, respectively.

## Discussion

In the present study, our results showed that APT MRI could detect ICH at hyperacute, acute and subacute stages. Moreover, APT MRI could provide additional information for the intracranial hemorrhage compared with SWI. Although the appearance of ICH in the APTw image may be not match with that in the SWI image, APT MRI could provide a new insight into investigate the contents of hematoma. Our promising findings indicate that the noninvasive APT MRI technique has the potential usefulness in the clinical identification of ICH at hyperacute, acute and subacute stages.

Our previous study in the rat models of strokes showed that the hyperacute ICH produced a consistently high APTw signal intensity relative to the contralateral normal brain tissue^16^. Our current results are consistent with the findings from the previous study. Furthermore, we found that the APTw signal intensity was consistently higher in the ICH at acute and subacute stages than that in the contralateral brain tissues. The increased APTw signal intensity in the hematoma at hyperacute, acute and subacute stages may be attributed to the abundant endogenous mobile proteins and peptides. The source of the mobile proteins and peptides may be the blood products in the hematoma. The hyperacute hematoma that forms after vessel rupture has high protein content because it consists of a collection of red blood cells, which are rich in hemoglobin, as well as white blood cells, platelet clumps, and protein-rich serum^17^. During the subsequent acute phase, the hematoma maintains high concentrations of protein because of the increased concentration of hemoglobin^17^. In this study, although the APTw signal intensity of the subacute hematoma was significantly higher than that of the contralateral normal brain tissue, it was significantly lower than that of the hyperacute hematoma. This finding indicated that the concentration of protein components in the subacute hematoma was less than that in the hyperacute hematoma, but still higher than that of the normal brain tissue. The progressive red blood cell lysis and proteolysis of the globin protein may result in a decreased concentration of protein content in the subacute hematoma^17^.

It is noteworthy that APTw signal intensity is also influenced by brain tissue pH^13^. The hematoma has higher pH in comparison with the normal brain tissue, which may further increase the APTw signal intensity^16^. Previous studies have shown that the cerebral ischemia at the hyperacute or acute stage produces the lower APTw signal intensity than the contralateral normal brain tissue because the pH of the ischemic lesion is decreased by the tissue acidosis^14^,^15^. The appearance of the APTw signal intensity during hyperacute and acute ischemia is the opposite of that produced by hemorrhagic stroke^16^. Thus, APT MRI has the potential to simultaneously detect and differentiate ICH from ischemic stroke at early stage in clinic.

The APT contrast visualized at around 3.5 ppm may include other contributions beside mobile proteins and peptides such as the nuclear Overhauser effect (NOE), amine and guanidinium chemical exchange-dependent saturation transfer signals^18^,^19^,^20^,^21^. The NOE due to the proteins in the hematoma may increase the APT image contrast between the ICH and the contralateral normal brain tissue^20^. However, the NOE can be clearly detected at lower saturation powers, while the APT_W_ signal is maximized at relatively higher saturation powers^20^. In this study, the saturation power of 3.0 μT was used to minimize the NOE effects for APT MRI. B_0_ inhomogeneity has big influences on the APT effect. Many methods have been used for B_0_ inhomogeneity corrections, including WASSAR and z-spectrum methods. In this study, we used a z-spectrum based B_0_ correction to assess the APT effect due to sufficient separation of the chemical exchange saturation transfer and water saturation curves as in the previous APT MRI studies using this method^11^,^12^. In addition, the NOE is generally ignorable at 3-T MRI, but it is clearly observed at ultrahigh magnetic fields^20^. Although the APT contrast may be contaminated by the amine and guanidinium chemical exchange-dependent saturation transfer signals at 2 ppm downfield from water, the contributions should be very small or negligible^21^.

SWI is sensitive to paramagnetic blood products, such as deoxyhemoglobin, methemoglobin, and hemosiderin, which are present in hematoma at different stages^3^,^9^,^10^. Our results showed that SWI could reliably identify ICH at hyperacute and acute stages. However, the SWI signal intensity did not differ significantly between the subacute hematoma and the contralateral normal brain tissue. This finding implies that the complicated constitution of subacute hematoma results in variable SWI signal intensity, which is influenced by many factors, such as water content and gliosis.

Our study has several limitations. First, the patient population was relatively small. We did not include patients with chronic ICH in this study because it is easy to diagnose this condition by conventional MRI scans. Second, the APT MRI used in this study was a 2-dimensional single-slice approach, which does not provide coverage of the whole ICH lesion. Third, we did not compare the ability of APT MRI and SWI to detect ischemic stroke. In the future, these two methods for identifying ischemic stroke should be compared, and the value of combining APT MRI and SWI should be investigated further. In addition, the values of APTw signal intensity in the prediction of ICH prognosis need to be further investigated.

In conclusion, APTw signal intensity may serve as a reliable, noninvasive imaging biomarker for detecting ICH at hyperacute, acute and subacute stages. Moreover, APT MRI could provide additional information for the intracranial hemorrhage compared with SWI.

## Methods

### Patient population

A total number of 42 patients with ICH (28 males and 14 females; age range, 22–86 years; mean age, 52 years) were enrolled in this study. ICH was confirmed in all patients by head CT within 2 days of stroke symptom onset because CT was considered the gold standard for imaging ICH^4^. The inclusion criterion of different stages of ICH was based on the interval between the stroke symptom onset and MRI examination. The patients were divided into hyperacute (interval not more than 12 hours), acute (interval, 13–72 hours), and subacute (interval, 73–720 hours) stages^8^. 9 patients were excluded because motion artifacts led to poor imaging quality. A total number of 33 patients with ICH were included in the statistical analysis. This study was approved by the institutional review board of Zhengzhou University People’s Hospital & Henan Provincial People’s Hospital, and written informed consent was obtained from each subject before participation. All methods were performed in accordance with the relevant guidelines and regulations.

### Image data acquisition

Head CT was performed on a 16-row CT scanner (Brilliance 16, Philips Medical Systems, Eindhoven, Netherlands) with a thickness of 5 mm, a tube voltage of 120 kV, and a tube current of 300 mA.

All patients underwent conventional MRI, SWI, and APT MRI on a 3-T MRI unit (Magnetom Trio, Siemens Medical Solutions, Erlangen, Germany) with a 12-channel head coil (Siemens Healthcare). The conventional MRI included an axial T2w image, an axial T1w image, an axial T2w dark-fluid image, and a sagittal T1w image. The T2w image was acquired using a turbo spin-echo (TSE) sequence with a slice thickness of 5 mm, a repetition time (TR) of 3500 ms, an echo time (TE) of 90 ms, a field of view (FOV) of 220 × 220 mm^2^, and a matrix of 256 × 256. The axial T1w image was obtained using a gradient echo (GRE) sequence (slice thickness, 5 mm; TR, 250 ms; TE, 2.48 ms; FOV, 220 × 220 mm^2^; and matrix, 256 × 256). A TSE sequence was performed on the T2w dark-fluid image (slice thickness, 5 mm; TR, 8500 ms; TE, 91 ms; FOV, 220 × 220 mm^2^; and matrix, 256 × 256). The sagittal T1w image was acquired using a GRE sequence (slice thickness, 6 mm; TR, 250 ms; TE, 2.48 ms; FOV, 240 × 240 mm^2^; and matrix, 320 × 256). The acquisition times for the axial T2w, T1w, T2w dark fluid and sagittal T1w images were 40 seconds, 66 seconds, 85 seconds and 46 seconds, respectively.

SWI is a 3-dimensional GRE sequence with a slice thickness of 2 mm, a TR of 30 ms, a TE of 20 ms, an FOV of 256 × 256 mm^2^, and a matrix of 512 × 512. The acquisition time was 252 seconds.

APT MRI was performed by using a prototype 2-dimensional single-slice radiofrequency-spoiled GRE protocol, with a slice thickness of 5 mm, a TR of 3200 ms, a TE of 2.87 ms, an FOV of 256 × 256 mm^2^, a matrix of 128 × 128, a flip angle of 12 degree, and 21 frequency offsets from +5 to −5 ppm. The presaturation was achieved using a train of five Gaussian-shaped radiofrequency saturation pulses with a continuous-wave amplitude equivalent of 3.0 μT. The length of the each saturation radiofrequency pulse was 99 ms and the interpulse delay was 100 ms. The total duration of the saturation pulse train was 995 ms followed by the readout of a single GRE image with centric reordering, leaving an effective TR of 4195 ms between consecutive frequency offset acquisitions. The conventional MRI was used to confirm the lesion position. Then, the two radiologists independently chose one slice with maximum lesion area from conventional MRI images for each patient. The slices of APT MRI were positioned at the same slice positions as the selected conventional MRI images. The acquisition time was 105 seconds for one slice of APT MRI.

### Image data processing and analysis

MRI data were obtained and transferred to a workstation (Syngo MR B17, Siemens Healthcare Solutions) for analysis. The B_0_ field inhomogeneity was calculated according to the deviation of the minimum of the fitted curve from 0 ppm. The APT images were calculated by the magnetization transfer asymmetry between signal intensities of ±3.5 ppm with respect to the water frequency using B_0_ corrected z-spectrum on a pixel-by-pixel basis^22^. The APT image was calculated by the following equation^23^:


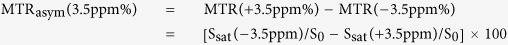


Where MTR_asym_ represents the magnetization transfer ratio asymmetry. MTR represents the magnetization transfer ratio. S_sat_ and S_0_ represent the signal intensities measured with and without radiofrequency saturation, respectively.

The MTR(3.5 ppm) image was calculated by the following equation^23^:


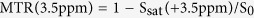


Where MTR represents the magnetization transfer ratio. S_sat_ and S_0_ represent the signal intensities measured with and without radiofrequency saturation, respectively.

The SWI images were created by using the magnitude and phase images^10^.

The two radiologists independently analyzed all MRI data. The region of interest of ICH lesion was hand-drew respectively on the APT and SWI image for the statistical analyses. The two radiologists independently hand-drew regions of interest that contained the whole ICH lesion on the APT and SWI images. The region of interest on the APT image was copied to the MTR(3.5 ppm) image of the same patient for the measurement. The regions of interest varied from 52 to 616 mm^2^ (mean area: 160 mm^2^). The mean values of APTw, MTR(3.5 ppm) and SWI signal intensities for each patient measured by the two radiologists were used for statistical analyses.

### Statistical analysis

All statistical analyses were performed with SPSS software (version 17.0; SPSS, Chicago, Ill). The APTw, MTR(3.5 ppm) and SWI signal intensities between the ICH and the corresponding contralateral normal brain tissue were compared by using a two-sided Wilcoxon signed-rank test. A two-sided Mann-Whitney *U* test was used to detect differences in APTw, MTR(3.5 ppm) and SWI signal intensities of ICH at hyperacute, acute and subacute stages. Bonferroni corrections were used for multiple comparisons. ROC curve analysis was used to assess the diagnostic utilities of APT MRI and SWI. We analyzed the AUCs to identify ICH at each stage based on APTw and SWI signal intensities. Interobserver agreements for the measurements of APTw, MTR(3.5 ppm) and SWI signal intensities were calculated by using intraclass correlation coefficients with 95% confidence intervals.

## Additional Information

**How to cite this article**: Ma, X. *et al*. Amide proton transfer magnetic resonance imaging in detecting intracranial hemorrhage at different stages: a comparative study with susceptibility weighted imaging. *Sci. Rep.* **7**, 45696; doi: 10.1038/srep45696 (2017).

**Publisher's note:** Springer Nature remains neutral with regard to jurisdictional claims in published maps and institutional affiliations.

## Figures and Tables

**Table 1:** APTw, MTR(3.5 ppm) and SWI signal intensities (mean ± standard deviation) in the ICH and contralateral normal brain tissue.

ICH stage	Normal brain tissue	Hematoma
Hyperacute ICH (n = 7)		
APTw signal (%)	1.2 ± 0.4	4.6 ± 0.8*
MTR(3.5 ppm) signal	0.70 ± 0.01	0.58 ± 0.07*
SWI	247.9 ± 28.8	142.8 ± 62.5*
Acute ICH (n = 7)		
APTw signal (%)	1.5 ± 0.4	3.8 ± 1.6*
MTR(3.5 ppm) signal	0.71 ± 0.01	0.54 ± 0.09*
SWI	249.9 ± 44.4	124.0 ± 66.7*
Subacute ICH (n = 19)		
APTw signal (%)	1.2 ± 0.3	3.2 ± 1.9*
MTR(3.5 ppm) signal	0.71 ± 0.02	0.54 ± 0.08*
SWI	243.2 ± 42.8	198.6 ± 149.4

^*^Significant between ICH and contralateral normal brain tissue by using a two-sided Mann-Whitney *U* test (p < 0.05); APTw, amide proton transfer-weighted; MTR, magnetization transfer ratio; ICH, intracranial hemorrhage; SWI, susceptibility weighted imaging.
